# Identification of immune subtypes and their prognosis and molecular implications in colorectal cancer

**DOI:** 10.1371/journal.pone.0278114

**Published:** 2022-11-23

**Authors:** Yan Sun, Hongping Li, Zhiming Ma, Jianfei Wang, Huiyu Yang, Xiaopeng Zhang, Bingrong Liu

**Affiliations:** 1 Department of Gastroenterology, The First Affiliated Hospital of Zhengzhou University, Zhengzhou, China; 2 Research and Development Division, Oriomics Biotech Inc, Hangzhou, Zhejiang, China; Universita degli Studi di Torino, ITALY

## Abstract

Immune composition is commonly heterogeneous and varies among colorectal cancer (CRC) patients. A comprehensive immune classification may act as important characteristics to predict CRC prognosis. Thus, we aimed to identify novel immune specific subtypes to guide future therapies. Unsupervised clustering was used to classify CRC samples into different immune subtypes based on abundances of immune cell populations, during which TCGA and GSE17536 datasets were used as training and validation sets, respectively. The associations between the immune subtypes and patient prognosis were investigated. Further, we identified differentially expressed genes (DEGs) between immune high and low subtypes, followed by functional enrichment analyses of DEGs. The expression levels of 74 immunomodulators (IMs) across immune subtypes were analyzed. As a result, we clustered CRC samples into three distinct immune subtypes (immune high, moderate, and low). Patients with immune-high subtype showed the best prognosis, and patients with immune-low subtype had the worst survival in both TCGA and GSE17536 cohorts. A group of 2735 up-regulated DEGs were identified across immune high and low subtypes. The main DEGs were the members of complement components, chemokines, immunoglobulins, and immunosuppressive genes that are involved in immune modulation-related pathways (e.g., cytokine-cytokine receptor interaction) or GO terms (e.g., adaptive immune response and T cell activation). The expression levels of 63 IMs were significantly varied across immune subtypes. In conclusion, this study provides a conceptual framework and molecular characteristics of CRC immune subtypes, which may accurately predict prognosis and offer novel targets for personalized immunotherapy through modifying subtype-specific tumor immune microenvironment.

## Introduction

Colorectal cancer (CRC) occurring from the colorectal epithelium is the third most common malignancy worldwide with 1.85 million new cases and 881 thousand deaths in 2018 [1]. CRC is a heterogeneous disease. CRC patients with different subtypes present widely variations in prognosis and drug responses to therapies [2]. Over the past few years, great efforts have been made to the classification of CRC subtype based on molecular profiles. At the genomic level, two major CRC phenotypes are previously recognized, one of which is microsatellite instability (MSI) phenotype that accounts for approximately 15% sporadic CRC and is responsible for the loss of DNA mismatch repair activity [3], whereas another chromosomal instability (CIN) phenotype that accounts for 85% of sporadic CRC, is characterized by gross chromosomal lesions [4]. In 2015, the international CRC subtyping consortium integrates six independent CRC classification systems to propose four consensus molecular subtypes (CMS) for CRC (MSI immune, canonical, metabolic, and mesenchymal) [5]. Despite current efforts on CRC classifications, identification of new CRC-specific subtypes may guide new directions for future therapies.

The development and progression of tumors are not only associated with aberrant proliferation of cancer cells, but also related to diverse components of tumor microenvironmental (TME) and tumor immune microenvironment (TIME) [6, 7]. Immune composition is commonly heterogeneous and varies among patients with the same tumor type, as well as affects the prognosis of cancer patients. Recently, Becht *et al*., [8] have demonstrated that the TME compositions are closely associated with different CRC subtypes. MSI immune subtype is featured by high levels of cytotoxic lymphocytes (CD8^+^ T cells and CD68 macrophages), and patients with this subtype exhibit good prognosis. Contrastively, mesenchymal subtype with poor prognosis reflects high infiltration of lymphocytes (activated CD8^+^ and natural killer (NK) cells) and high density of fibroblasts. In addition, based on the heterogeneity of 22 immune cells in CRC tissues across different tumor stages, an immune-risk score model has been constructed to predict prognosis of CRC [9]. A new CRC subtyping based on immune landscapes of CRC patients may act as an important method to predict prognosis. However, such immune classification is still challenging and limited in CRC.

Compared with traditional cancer classifications, the recent immune classification has advantages in considering the wide heterogeneity of TME regardless of tumor location or histology. Up to date, immune classifications have been applied to more precisely characterize CRC subtypes through estimating and distinguishing immune cell abundance among previously identified molecular subgroups [8, 10]. For example, Tang *et al*. have classified CRC patients into four immune subtypes according to the levels of T-cell populations [11]. Despite such previous efforts, no immune subtyping on the basis of the overall abundance of immune cell populations has been independently investigated in CRC. In this study, we identified three immune-specific subtypes of CRC based on the abundance of eight immune cell populations. We demonstrated the capability of the immune subtypes in differentiating prognosis of CRC patients, although this study did not aim to develop a real classifier for CRC, due to the unsupervised clustering approach we used. Furthermore, we identified differentially expressed genes (DEGs) between immune high and low subtypes, and explored the potential functions of these DEGs.

## Methods

### Public transcriptomic data acquirement and preprocessing

The RNA-sequencing data of 433 colon adenocarcinoma (COAD) samples and related clinical information including age, sex, stage, vital status, and time to last contact were downloaded from The Cancer Genome Atlas (TCGA, https://portal.gdc.cancer.gov/) as the training set. Transcriptome profiles and clinical information of 177 CRC samples from GSE17536 (https://www.ncbi.nlm.nih.gov/geo/query/acc.cgi?acc=GSE17536) dataset were acquired from GEO database as the validation set. The profiles of GSE17536 were produced on the platform of GPL570 [HG-U133_Plus_2] Affymetrix Human Genome U133 Plus 2.0 Array.

The normalized expression data of samples from TCGA were the Fragments Per Kilobase of transcript per Million with upper quartile normalization (FPKM-UQ) type. Series Matrix File(s) of the GSE17536 normalized data were downloaded, in which the raw data had been normalized by the robust multiarray average (RMA) method and processed by Bioconductor’s affy package, including background correction and quantile normalization [12].

### Immune stratifications and subtype validation

The microenvironment cell populations-counter (MCP-counter) tool can robustly quantify the abundance of eight immune cells populations (T cells, NK cells, neutrophils, myeloid dendritic cells, monocytic lineage, cytotoxic lymphocytes, CD8+ T cells, and B cell lineage) and two stromal populations (endothelial cells and fibroblasts) of TME based on the expression of cell type-specific gene signatures [13]. In our study, in order to build a robust immune classification of CRC, we firstly evaluated the abundances of eight immune cell populations of samples from the TCGA and GSE17536 dataset using the MCP-counter tool (version 1.2.0), individually; and the two stromal populations that exhibited high heterogeneity with their signature expression across all samples were removed. Then, the abundances of eight immune cell populations were normalized using the Z-score method, resulting in similar distributions of the abundances across samples. Unsupervised clustering of samples in the TCGA cohort was performed to investigate the immune subtypes of CRC using the K-means clustering algorithm. Based on gathered clusters, the CRC samples were classified into different immune infiltration subtypes. To validate the immune subtypes identified from the training set, for each samples in GSE17536 dataset, we calculated distances between the abundance of eight immune cells and mean abundances of the same type of immune cells in each TCGA subtypes. The samples in GSE17536 dataset were classified according to their similarity (minimum distance) to TCGA subtypes. The heatmaps were plotted by the “pheatmap” package (version 1.0.12) in R.

### Prognostic associations of immune subtypes

To assess the prognostic associations of immune subtypes with overall survival (OS), CRC patients with > 120 months follow-up in the TCGA and GSE17536 cohorts were used for internal test and external validation, respectively. Kaplan-Meier analysis was conducted to explore the prognostic associations of immune subtypes using the “survival” package (version 3.1–11) in R and the Kaplan-Meier curves were plotted by the “survmine” package (version 0.4.6). The differences in OS among different subtypes were detected by the log-rank test and a *P* < 0.05 was considered statistically significant. Multivariate Cox model was used to evaluate the independent prognostic value of immune subtypes using the coxph function in “survival” package.

### Identification of DEGs between immune high and low subtypes and functional enrichment analysis

In order to explore the molecular differences between immune high and low subtypes, the DEGs analysis from the TCGA database was conducted using the DESeq2 function in R package. Briefly, the differential expression of genes between immune high and low subtypes was compared using a *t* test and the *P* values were revised by Benjamin-Hochberg (BH). The thresholds of |Log_2_ fold change (FC)|>1 and *P*adj<0.05 were applied for screening DEGs.

To further analyze the biological functions of DEGs, the functional enrichment of Gene Ontology (GO) terms comprised of biological process (BP), cellular component (CC), molecular function (MF), and Kyoto Encyclopedia of Genes and Genomes (KEGG) pathway of up-regulated and down-regulated DEGs were performed by the “clusterProfiler” (version 3.12.0) package in R [14]. The GO and KEGG items with a *P* < 0.05 were considered statistically significant.

### Immunomodulator (IM) expression analysis across immune subtypes

A previous study provided a group of 78 IMs curated from literature reviews and with immunomodulatory functions confirmed by immuno-oncology experts [15]. In this study, we excluded 4 of 78 IMs (*HLA-DRB*3, *HLA-DRB4*, *VEGFB*, and *KIR2DL2*) since their expression data were unavailable. Thus, we analyzed the differences in 74 IMs expression from the TCGA cohort across immune subtypes. The mean expression levels of each of the 74 genes were calculated using the expression data of all the patients’ samples in each subtypes, and compared among three subtypes using ANOVA test. A *P* value of < 0.05 was considered statistically significant.

## Results

### TME-based compositions classify CRC into three immunes subtypes

**Fig 1:**
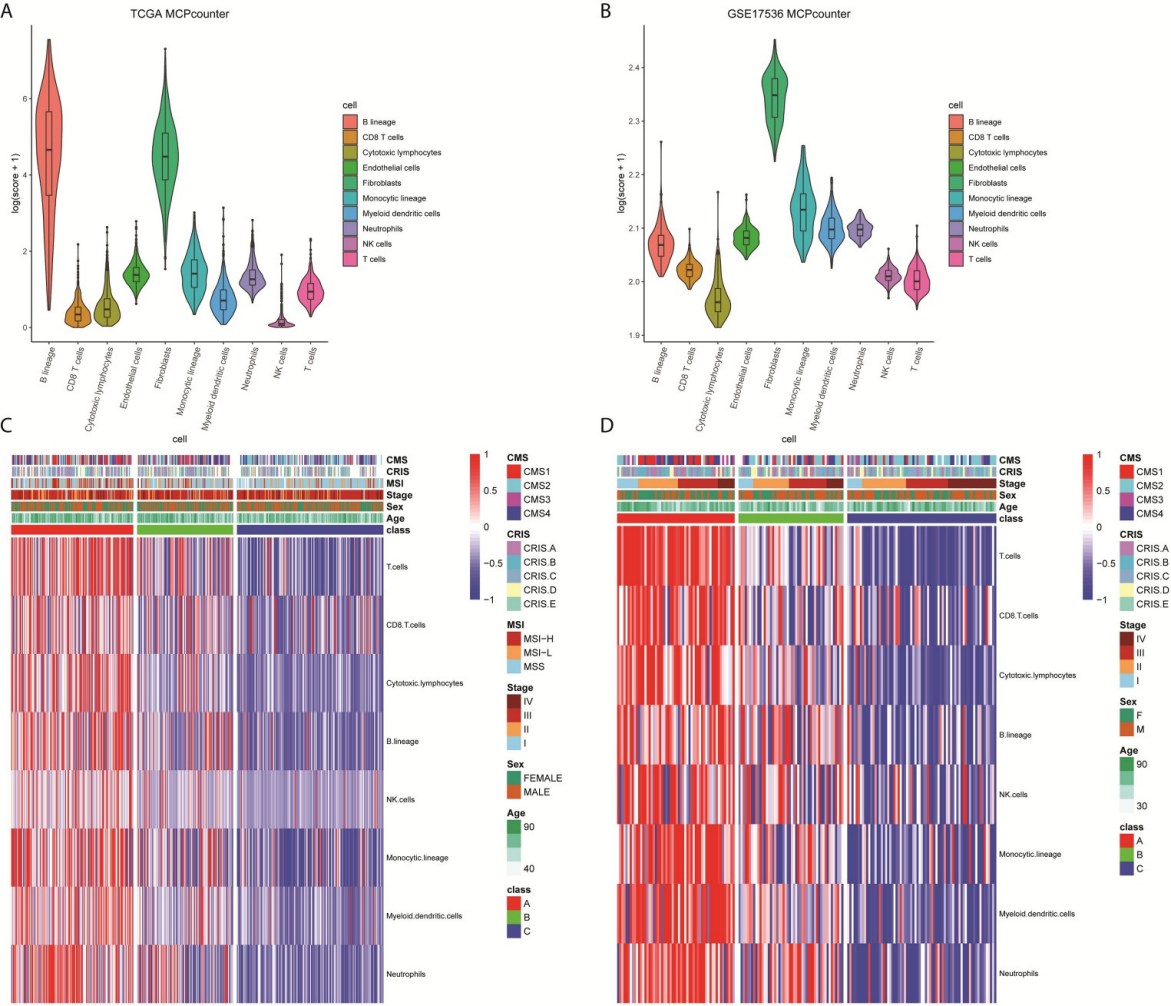
Immune classification of CRC based on the immune infiltrating cells populations. The violin-plot showed the abundances of distinct cell populations using samples from the TCGA cohort (**A**) and GSE17536 cohort (**B**). The violin-plots were plotted by ggplot2 (version 3.3.0) in R. Unsupervised clustering of samples from the TCGA training set (**C**) and the GSE17536 validation set (**D**) revealed that three distinct immune subtypes were clustered: immune-high (subtype A, red), immune-medium (subtype B, green), and immune-low (subtype C, dark blue). The color changes from red to dark blue stand for cell population abundances from high to low. In addition to three immune subtypes identified in this study, CMS subclasses, CRIS subclasses, status of microsatellite instability, and patient demographic/clinical characteristics (age, sex, and tumor stage) were also plotted for each samples. CRC: colorectal cancer; TCGA: The Cancer Genome Atlas; CMS: consensus molecular subtype; CRIS: CRC intrinsic subtype; MSI-H: microsatellite instability high; MSI-L: microsatellite instability low; MSS: microsatellite stable.

In order to build immune classification of CRC, the TME compositions of samples from the TCGA and GSE17536 datasets were analyzed. First, distinct abundances of immune and stromal cell populations of samples were evaluated (**Fig 1A and 1B**). Thus, the abundances of eight immune cell populations (T cells, CD8^+^ T cells, cytotoxic lymphocytes, B lineage, NK cells, monocytic lineage, myeloid dendritic cells, and neutrophils) were normalized using Z-scores. Unsupervised clustering analysis of TCGA samples (training set) revealed that CRC samples were clustered into three distinct immune subtypes, labeled A, B, and C (**Fig 1C**).

The subtype classifier defined by the TCGA training set was then validated using the GSE17536 dataset. Similarly, the samples were divided into three immune subtypes (**Fig 1D**). We further compared the differences of immune cell populations across three subtypes in the training and validation datasets, and found that subtype A (immune-high) was consistently characterized by the highest abundances of eight immune cells, whereas subtype C (immune-low) was consistently characterized by the lowest abundances of these cells.

Recent studies have reported several CRC classifiers based on transcriptional signatures, for example, CMS [5] and CRC intrinsic subtypes (CRIS) [16]. To assess the similarity and difference between our subtypes and these published classifiers, we determined CMS and CRIS subclasses for each samples. As shown in **Fig 1C**, **1D**, and **Table 1**, in both training and validation datasets, CMS1 and CRIS-A were enriched in immune-high subtype A. CMS1 samples were reported to be hypermutated and encompass the majority of MSI tumors [5]. Similarly, CRIS-A was considered an MSI-like, *BRAF*- or *KRAS*-mutated, and secretory subtype. With the available whole exome sequencing data and MSI status from TCGA, we evaluated mutational signatures across three immune subtypes, and found that somatic mutations and MSI-H samples also enriched in subtype A (**S1 Fig**). Specifically, samples with mutated *BRAF* were predominantly assigned to subtype A (*P* < 0.001). Of note, our subtypes based on abundances of immune cell populations were significantly different from existing transcriptional subtypes or genomic markers (**Table 1**), although some overlapping was observed, thus offering complementary information.

**Table 1:** Distributions of immune subtypes.

		TCGA				GSE17536			
		A	B	C	*P*	A	B	C	*P*
		N (%)	N (%)	N (%)		N (%)	N (%)	N (%)	
**CMS**	**CMS1**	43 (29.7)	18 (15.8)	6 (3.5)	<0.0001	19 (33.9)	8 (16.0)	4 (5.6)	<0.0001
	**CMS2**	33 (22.8)	33 (29.0)	72 (41.4)		7 (12.5)	19 (38.0)	39 (54.9)	
	**CMS3**	8 (5.5)	17 (14.9)	28 (16.1)		7 (12.5)	5 (10.0)	8 (11.3)	
	**CMS4**	34 (23.5)	19 (16.7)	31 (17.8)		16 (28.6)	10 (20.0)	14 (19.7)	
	**NA**	27 (18.6)	27 (23.7)	37 (21.3)		7 (12.5)	8 (16.0)	6 (8.5)	
**CRIS**	**A**	38 (26.2)	6 (5.3)	28 (16.1)	<0.0001	20 (35.7)	14 (28.0)	12 (16.9)	0.020
	**B**	21 (14.5)	9 (7.9)	8 (4.6)		17 (30.4)	7 (14.0)	10 (14.1)	
	**C**	26 (17.9)	21 (18.4)	32 (18.4)		8 (14.3)	12 (24.0)	24 (33.8)	
	**D**	15 (10.3)	14 (12.3)	19 (10.9)		6 (10.7)	8 (16.0)	9 (12.7)	
	**E**	9 (6.2)	20 (17.5)	30 (17.2)		5 (8.9)	8 (16.0)	16 (22.5)	
	**NA**	36 (24.8)	44 (38.6)	57 (32.8)		0 (0)	1 (2.0)	0 (0)	
**MSI**	**MSI-H**	44 (30.3)	17 (14.9)	10 (5.8)	<0.0001	-	-	-	-
	**MSI-L**	24 (16.6)	21 (18.4)	31 (17.8)		-	-	-	
	**MSS**	71 (49.0)	69 (60.5)	121 (69.5)		-	-	-	
	**NA**	6 (4.1)	7 (6.1)	12 (6.9)		-	-	-	
***BRAF***	**Mut**	35 (24.1)	12 (10.5)	10 (5.7)		-	-	-	
	**Wild**	95 (65.5)	92 (80.7)	158 (90.8)	<0.001	-	-	-	
	**NA**	15 (10.3)	10 (8.8)	6 (3.4)		-	-	-	
***KRAS***	**Mut**	45 (31.0)	41 (36.0)	91 (52.3)		-	-	-	
	**Wild**	85 (58.6)	63 (55.3)	77 (44.3)	0.002	-	-	-	
	**NA**	15 (10.3)	10 (8.8)	6 (3.4)		-	-	-	
**Age**	**≤65 y**	60 (13.9)	42 (9.7)	81 (18.7)	0.26	21 (11.9)	24 (13.6)	38 (21.5)	0.20
	**>65 y**	85 (19.6)	72 (16.6)	93 (21.5)		35 (19.8)	26 (14.7)	33 (18.6)	
**Sex**	**Female**	69 (47.6)	54 (47.4)	77 (44.3)	0.80	34 (60.7)	20 (40.0)	27 (38.0)	0.024
	**Male**	76 (52.4)	60 (52.6)	97 (55.7)		22 (29.3)	30 (60.0)	44 (62.0)	
**Stage**	**I**	5 (3.5)	4 (3.5)	3 (1.7)	0.30	10 (17.9)	7 (14.0)	7 (9.9)	0.22
	**II**	30 (20.7)	19 (16.7)	26 (14.9)		19 (33.9)	17 (34.0)	21 (29.6)	
	**III**	99 (68.3)	79 (69.3)	118 (67.8)		19 (33.9)	18 (36.0)	20 (28.2)	
	**IV**	11 (7.6)	12 (10.5)	27 (15.5)		8 (14.3)	8 (16.0)	23 (32.4)	

CMS: consensus molecular subtype; CRIS: CRC intrinsic subtype; MSI-H: microsatellite instability high; MSI-L: microsatellite instability low; MSS: microsatellite stable; Mut: mutant; NA: not available.

### CRC immune subtypes are associated with patient survival

**Fig 2:**
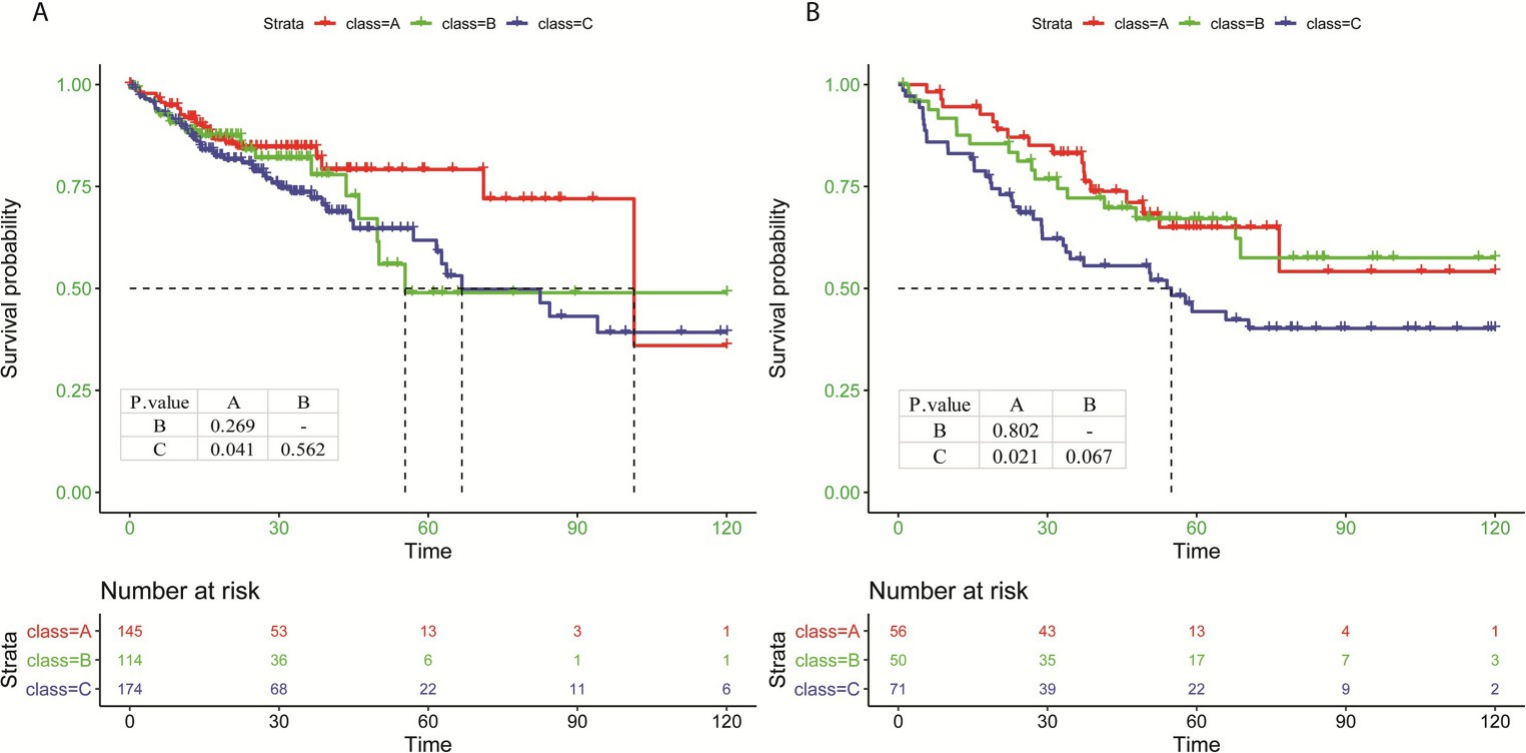
Prognostic stratification from immune subtypes. Kaplan–Meier curves for 10-year overall survival of patients in the immune-high (subtype A, red), immune-medium (subtype B, green), and immune-low (subtype C, dark blue) subtypes from the TCGA (**A**) and GSE17536 (**B**) cohorts. The X-axis represents the survival time counted by months, and Y-axis represents the survival probability of corresponding subtype. Dotted lines represent median survival time and survival probability of corresponding subtype.

To assess the associations between the immune subtypes and prognosis of CRC patients, the Kaplan-Meier analysis was conducted. A borderline but not significant difference in OS was observed among three immune subtypes in the TCGA cohort (*P* = 0.13). Among the three groups, CRC patients with subtype A had the best prognosis, while those with subtype C had the worst outcome (subtype A *vs*. C, *P* = 0.041, **Fig 2A**). Meanwhile, there was a significant difference in OS among three immune subtypes defined by the GSE17536 validation cohort (*P* = 0.034), showing patients in subtype A had longer OS than patients in subtype C (subtype A *vs*. C, *P* = 0.021, **Fig 2B**). The results from multivariate Cox model showed that after adjustment for age, sex, and tumor stage, death risk in patients with subtype C increased by 44% (hazard ratio (HR) = 1.44, *P* = 0.15) in the TCGA dataset and 92% (HR = 1.92, *P* = 0.039) in the GSE17536 dataset, as compared to patients with subtype A (**S2 Fig**). The consistently observed prognosis pattern in the training and validation datasets suggested the prognostic value of immune subtypes in CRC.

Immunoscore has been constructed to predict CRC prognosis using algorithms such as CIBERSORT [17] and ESTIMATE [18]. Previous studies reported better survivals in CRC patients with a low immunoscore or in an immune-activated subclass. To compare the findings of ours vs others, we calculated immunoscores using a 13 immunocyte-based prognostic model that was developed by implementing LASSO-Cox regression on the composition of 22 immune cell types estimated by CIBERSORT algorithm [17]. **S3 Fig** shows the distribution of immunoscores across three subtypes. In both training and validation datasets, subtype A that was associated with favorable outcome exhibited significantly lower immunoscores than subtype C (*P* < 0.001, and = 0.02, respectively). Thus, our study further demonstrated the prognostic value of immune-specific signatures, no matter which kind of algorithms was used to indicate an immune-activated subtype.

### Identification of DEGs between subtypes A and C and functional annotation

**Fig 3:**
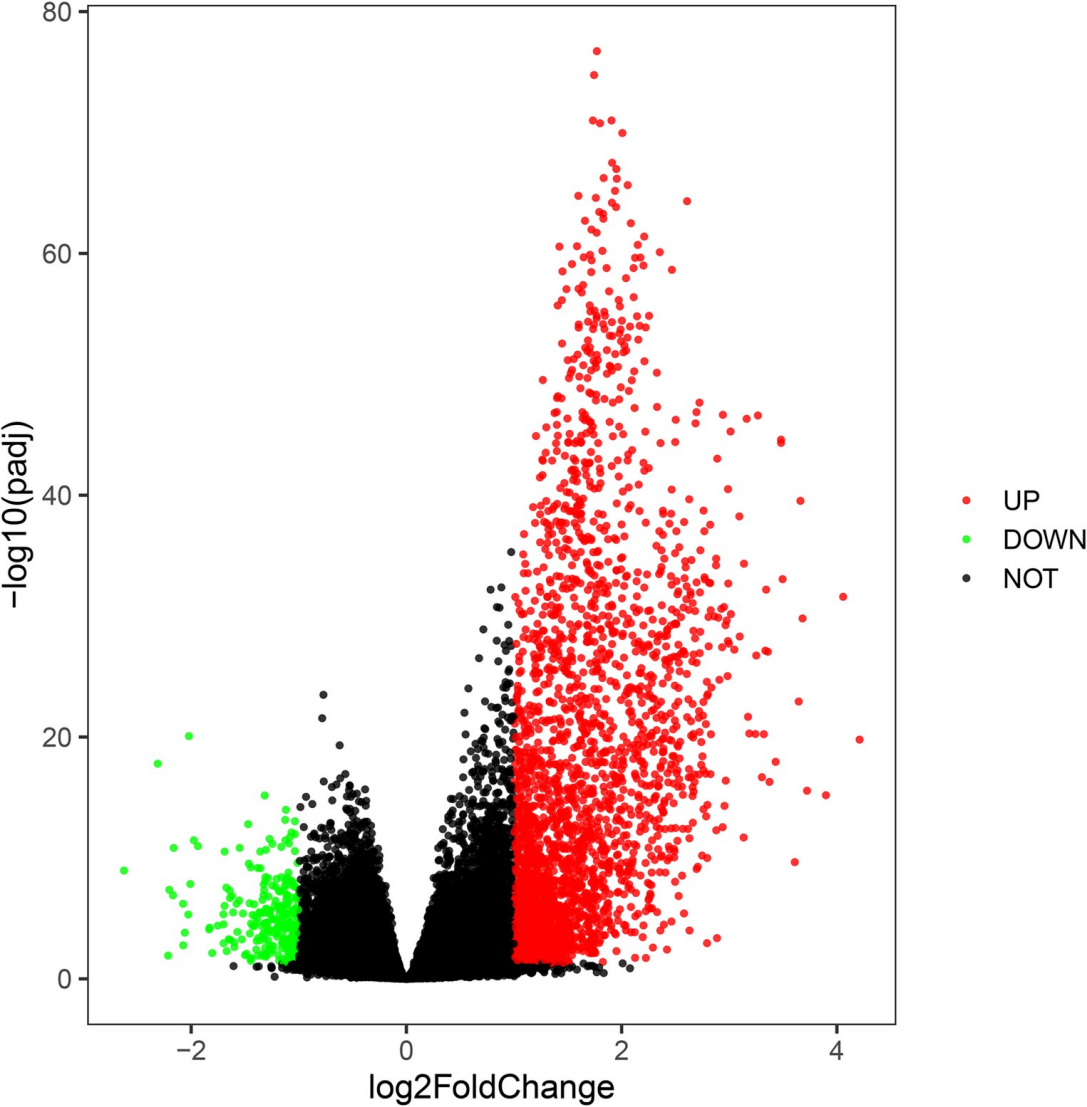
Volcano plot to show differentially expressed genes (DEGs) between immune-high (subtype A) and immune-low (subtype C) subtypes. The volcano plot was plotted by the ggplot2 package in R. The up-regulated DEGs are labeled by red dots, down-regulated DEGs are labeled by green dots, while non-DEGs are marked by dark dots. The X-axis denotes the log_2_ fold changes of expression ratios of corresponding genes, and the Y-axis denotes the *P* values of corresponding genes between subtypes A and C.

In sight of the significant difference in OS between subtypes A (immune-high) and C (immune-low), we further explored the molecular differences between these two subtypes and aimed to decipher the underlying mechanisms of survival differences at the molecular level. Based on the thresholds of |log_2_ FC|>1 and Padj<0.05, a group of 2735 up-regulated DEGs and 253 down-regulated DEGs were identified in subtype A, when compared to subtype C (**Fig 3**). The number of up-regulated DEGs was 10.8 times higher than that of down-regulated DEGs, which potentially confirmed the immune activation of subtype A. Subtype A exhibited high expression levels of complement components (e.g., *CFH*, *C7*, *C3*, *C1QC*, *C3AR1*, *C1QB*, *C1QA*, and *C5AR1*), chemokines (e.g., *CCL1*, *CCL2*, *CCL3*, *CCL3L1*, *CCL4*, and *CCL4L2*), immunoglobulins-related encoding genes (e.g., *IGLV3-22*, *IGKV1-39*, *IGLJ6*, *IGHJ2*, *IGLJ2*, and *IGLC7*), and immunosuppressive genes (e.g., *TGFB1*, *CD274*, *PDCD1* and *PDCD1LG2*).

**Fig 4:**
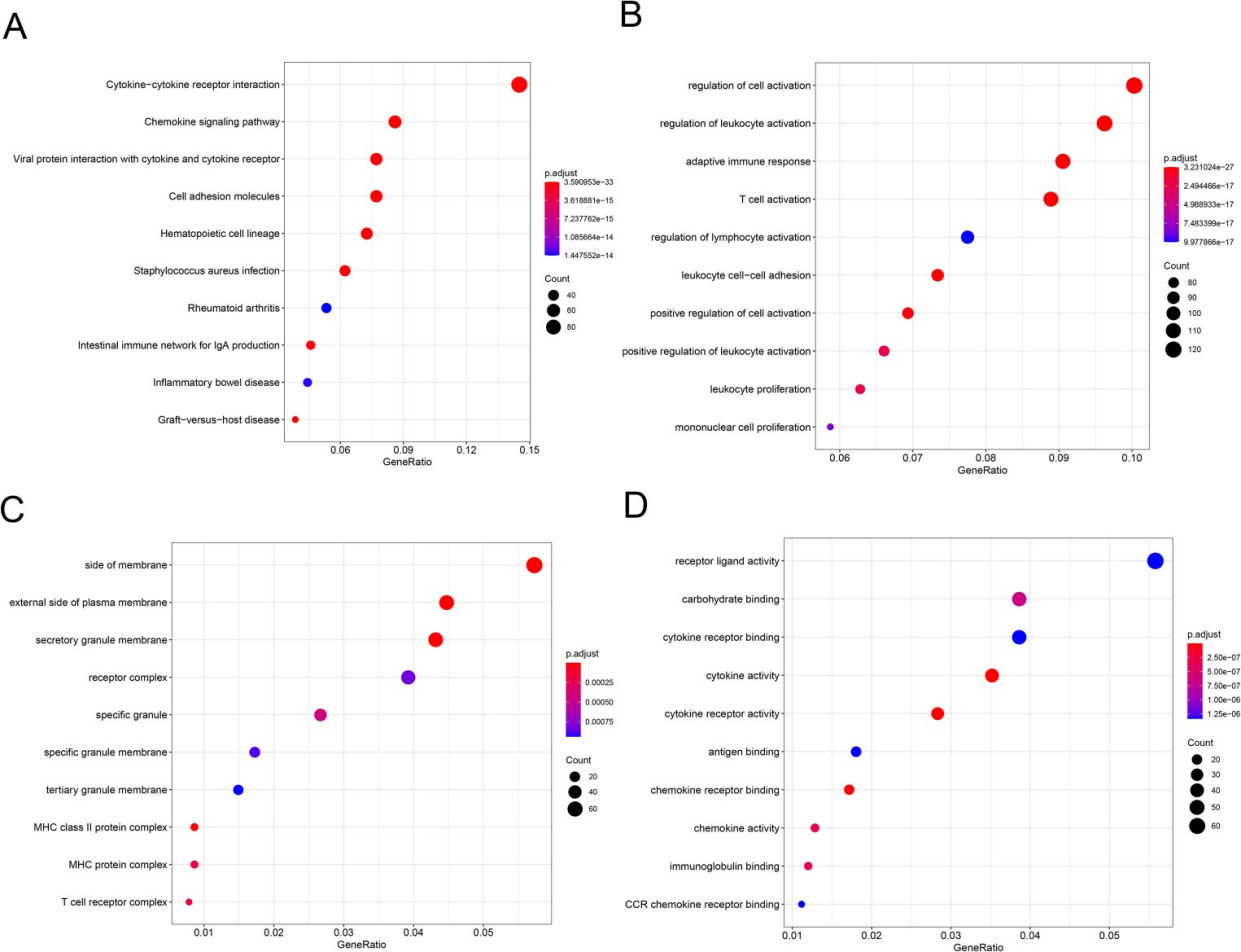
Functional enrichment analyses of up-regulated DEGs between subtypes A and C. The enrichment results in terms of KEGG pathways (**A**), Biological Process (**B**), Cellular Component (**C**), and Molecular Function (**D)** of GO terms. The size of a bubble is proportional to counts of genes enriched in corresponding functions. The color changes from red to dark blue denote the *P* value of corresponding functions from low to high.

We further investigated the biological functions of these DEGs between subtypes A and C. The functional enrichment results revealed that most up-regulated DEGs were associated with pathways of cytokine-cytokine receptor interaction (KEGG: hsa04060; P.adj = 3.59E-33), hematopoietic cell lineage (KEGG: hsa04640; P.adj = 2.61E-25), intestinal immune network for IgA production (KEGG: hsa04672; P.adj = 3.89E-20), GO terms of adaptive immune response (GO-BP:0002250, P.adj = 3.23E-27), regulation of leukocyte activation (GO-BP:0002694, P.adj = 1.21E-21), T cell activation (GO-BP:0042110, 8.59E-20), leukocyte proliferation (GO-BP:0070661, P.adj = 2.34E-17), mononuclear cell proliferation (GO-BP:0032943, P.adj = 8.42E-17), MHC protein complex (GO-CC:0042611, P.adj = 0.000186), T cell receptor complex (GO-CC:0042101, P.adj = 0.000202), cytokine activity (GO-MF:0005125, P.adj = 7.70E-10), and immunoglobulin binding (GO-MF:0019865, P.adj = 3.11E-07) (**Fig 4)**. These functions were all closely involved in immune activation. However, no associated KEGG pathway and GO term was enriched from down-regulated DEGs.

### IM gene expression across immune subtypes

**Fig 5:**
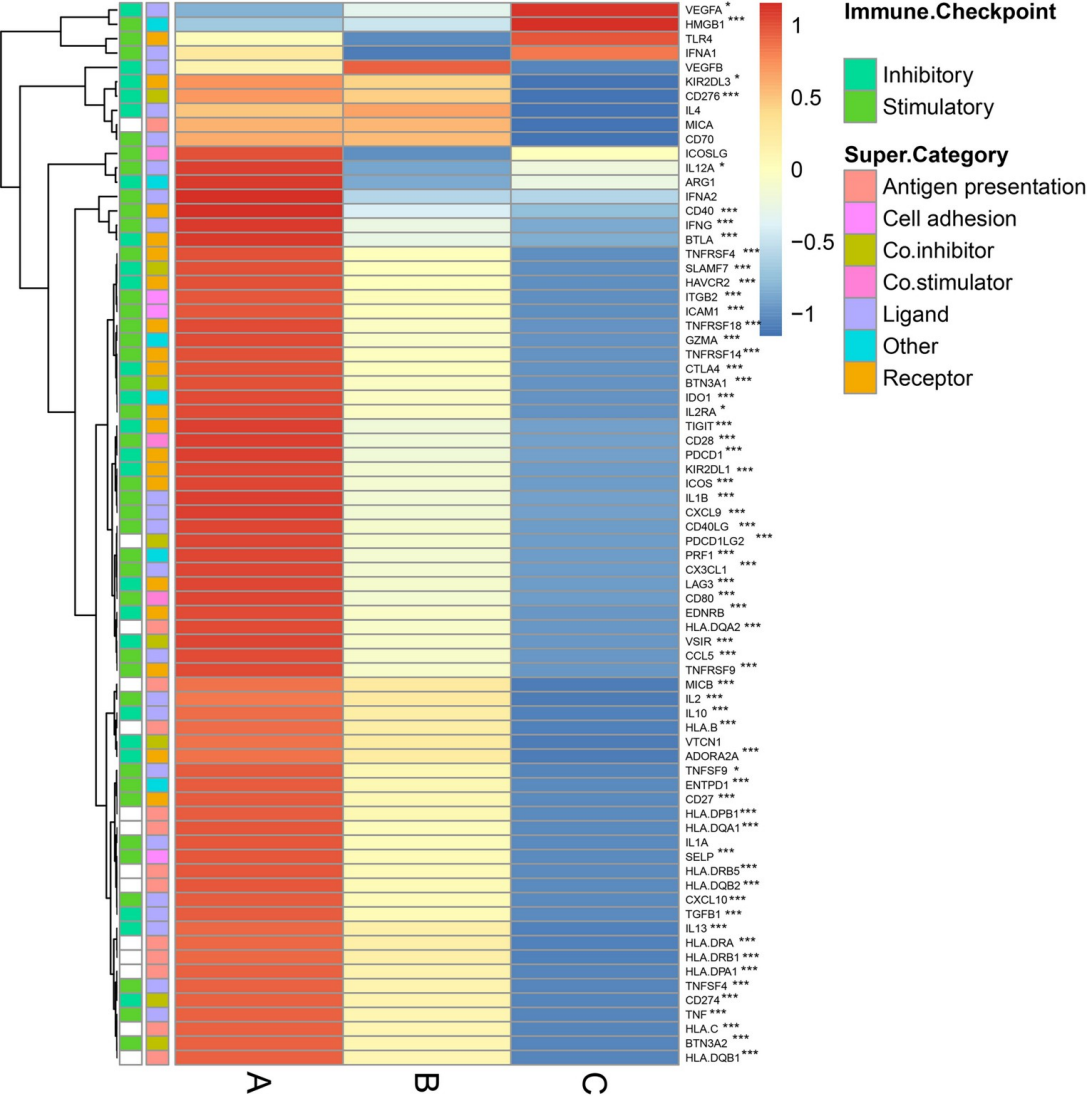
The heatmap shows differences in IM gene expression across immune subtypes. The color changes from red to dark blue denote the expression levels from high to low. The X-axis denotes the expression of IM in each subtype, and the Y-axis denotes the category of corresponding gene. IMs: immunomodulators. * *P* < 0.05, *** *P* < 0.01.

IM molecules have pivotal roles in cancer immunotherapy through modifying TME using numerous IM agents [19, 20]. Thus, understanding the difference of IM gene expression across immune subtypes is needed to uncover the immune response of CRC patients and provide potential targets for personalized immunotherapy. In our work, the expression levels of 74 IMs across three immune subtypes were analyzed using the TCGA database. Notably, we found that the expression levels of IMs varied across immune subtypes, and perhaps IM expression played important roles in determining immune subtypes through shaping the TME (**Fig 5**). Totally, the expression levels of 63 IMs were highly heterogeneous across three immune subtypes, and among whom, 59 IMs showed high expression in subtype A, moderate expression in subtype B, and low expression in subtype C (**Fig 5**); whereas the expression levels of the remaining four genes (*VEGFA*, *HMGB1*, *KIR2DL3*, and *CD276*) were highest in subtype C, followed by subtype B, and lowest in subtype A. Genes with the most significant differences across subtypes included *ICOS*, *IL2RA*, *SLAMF7*, *CTLA4*, and *TNFRSF9*. In addition, immune-checkpoint-related genes (e.g., *PDCD1*, *PDCD1LG2*, *CD274*, *CTLA4*, *CD276*, and *LAG3*), T-cell attracting chemokines (e.g., CXCL9 and CXCL10), major histocompatibility complexes (e.g., *HLA*.*DPB1*, *HLA*.*DRA*, *HLA*.*DPA1*, *HLA*.*DRB1*, *HLA*.*DQA1*, *HLA*.*DQB1*, *HLA*.*DQB2*, *HLA*.*DQA2*, and *HLA*.*DRB5*), and the interleukin (IL) familiarly members (e.g., *IL10*, *IL2*, *IL13*, *IL1B*, and *IL12A*) were all differentially expressed across subtypes. Therefore, the highest expression of these bulk IMs in subtype A and lowest in subtype C accounted for a descending order of immune activity of samples from subtype A to C. These molecular results are likely responsible for survival differences of subtypes and future validation of immune subtypes are required.

## Discussion

Immuno-oncology, as a novel emerging field, changes the standard of cancer treatment based on effective antitumor immune responses [21]. A good understanding of tumor-dependent heterogeneity of TME is needed for developing new immunotherapies to improve clinical outcomes of CRC patients. In this study, we proposed a classification of CRC into three distinct immune subtypes (immune high, moderate, and low). We found that the immune subtypes were associated with OS of CRC patients, and those with an immune-high subtype had the best survival. Moreover, the findings in the TCGA cohort were validated using the GSE17536 dataset. We further explored the differences between immune-high and immune-low subtypes at the molecular levels, and identified a group of 2988 DEGs including members of complement components, chemokines, immunoglobulins, and immunosuppressive genes that are involved in immune modulation-related pathways or GO terms. Additionally, a group of 63 IMs that heterogeneously expressed across three immune subtypes were found. This study provides a conceptual framework and molecular characteristics of CRC immune subtypes, which may accurately predict prognosis and offer novel targets for personalized immunotherapy through modifying subtype-specific TME.

The impacts of TME on survivals of cancer patients have been well investigated, and tumor-infiltrating immune cells have been associated with prognosis of multiple cancer types [22, 23]. It has been reported that CRC patients with a high density of CD8^+^ T cells have better survival than those with a low density of CD8^+^ T cells [24]. Particularly, tumor-infiltrating lymphocytes, including central memory CD8^+^ T cells, cytotoxic cells, NK cells, and B cells, are associated with prolonged prognosis of CRC, whereas several subpopulations like Th17 and mast cells are associated with shorter survival of CRC [25, 26]. Notably, patient survival is revealed to be associated with immune subtypes. For example, an immune-hot subtype of squamous cell carcinoma with the highest infiltration of CD8^+^ T cells and activated NK cells has the best prognosis, while the immune-cold subtype with the least lymphocyte infiltration demonstrates the worst prognosis [27]. A key observation by Angelova *et al*. has demonstrated that immune clustering can predict prognosis of stage I/II CRC patients [11]. Recently, Xiong *et al*. have classified CRC samples into high, medium, and low immune subtypes based on 29 immune-related items and revealed distinct survival patterns of these subgroups [28]. In line with these findings, we found that the immune-high subgroup presenting the highest infiltration of lymphocytes had the best prognosis among the three immune subtypes, revealing that the immune classification may serve as a complementary approach for prognosis prediction.

MSI is the hallmark of lynch syndrome (hereditary nonpolyposis colorectal cancer) and constitutional mismatch repair deficiency, and has been recommended by EMSO as a predictor for immunotherapy [29]. Tumor mutational burden is also an emerging tumor-agnostic biomarker for response to immunotherapy [30]. A high percentage of concordance of MSI-H and TMB-H was observed in cancers including CRC [29]. Previously reported CRC subclasses enriched for MSI or somatic mutations such as CMS1 and CRIS-A were predominantly assigned to subtype A in this study. Although different algorithms were utilized to analyze gene expression data, our study observed similar MSI/mutational status and clinical outcome in patients with this immune-high subtype. Meanwhile, it should be noted that MSI/MSS samples were not equally partitioned across the three immune subtypes. Zhang *et al*. recently reported that MSI-H was differentially associated with tumor immune microenvironment, and MSI-H CRC exhibited significantly altered immune phenotype, including an increase of CD8 T cells and alteration of CD4 functional subsets in favor of immune protection [31]. Therefore, the subtypes we developed based on abundances of immune cell populations can provide information complemental to other transcriptional classifiers or genomic markers, which may assist in identifying patients with immune hot tumors who are likely to benefit from immunotherapy.

Since in-depth analyses of the heterogeneity of CRC subtypes related to TIME at the molecular level is still insufficient, DEGs between immune-high and immune-low subtypes and potential functions of these DEGs were investigated in this work. As a result, a set of 2988 DEGs encoding members of complement components, chemokines, immunoglobulins, and so on were identified. Interestingly, the functional enrichment analyses revealed the involvement of up-regulated DEGs in immune modulation-related pathways or GO terms such as “cytokine-cytokine receptor interaction”, “hematopoietic cell lineage”, “intestinal immune network for IgA production”, “adaptive immune response”, “regulation of leukocyte activation and proliferation” [32], and “T cell activation” [33]. Consistently, Bech *et al*. have suggested the “hematopoietic cell lineage” pathway is overrepresented in C2 and C4 subtypes of CRC, and those two subtypes are all characterized with highly infiltrated immune cells [10]. Thus, the DEGs identified in the present study might account for shaping the distinct immune landscapes of TME between immune-high and immune-low subtypes.

Immunotherapy comprising the use of immunomodulatory antibodies, vaccines, and oncolytic viruses becomes a promising approach for cancer treatment [34]. A better understanding of the molecular mechanisms of immune regulation may provide opportunities for cancer immunotherapy. IMs are kinds of molecules that play crucial roles in immune regulation. In this study, we found 63 IMs involving immune-checkpoint-related genes (e.g., *PDCD1*, *PDCD1LG2*, *CD274*, *CTLA4*, *CD276*, and *LAG3*), T-cell attracting chemokines (e.g., *CXCL9* and *CXCL10*), and major histocompatibility complexes, class II (e.g., *HLA-DPB1*, *HLA-DRA*, *HLA*.*DRB1*, and *HLA-DQB1*) that were significantly heterogeneously expressed across three immune subtypes. Similarly, it has been revealed that consensus molecular subtype 1 of CRC characterized by high immune infiltration and activation has high expression of *CXCL9*, *CXCL10*, *CXCL13*, *HLA I*, *HLA II*, and immune checkpoint inhibitors [35]. Especially, *CXCL9* and *CXCL10*, known as T cell chemokines, are related to immune activation of T and NK cells [36]. HLA class II molecules like *HLA-DPB1*, *HLA-DRA*, *HLA-DRB1*, and *HLA-DQB1* are associated with antigen processing and presentation to CD4^+^ T cells, and the down-regulation of these genes is related to poor prognosis of cancers [37, 38]. Of note, in our study, *CXCL9*, *CXCL10*, and HLA class II molecules were up-regulated in the immune-high subtype, suggesting strong antitumor immunity and immune activation. However, highly expressed genes such as *CD274* (PD-L1), *PDCD1LG2* (PD-L2), and *CTLA4* in this subtype suggested treatment options using *PD-1* and *CTLA4* blockade therapies. In contrast, the immune-low cluster might require immune agonists or other approaches to restore T-cell activation. The immune subtyping of CRC in this study may reveal different strategies in guiding personalized immunotherapy.

## Conclusions

In sum, our study effectively classified CRC patients into three distinct immune subtypes: immune high, moderate, and low subtypes. The subtyping strategy may accurately stratify patients into different prognostic groups. The investigation of DEGs revealed the molecular characteristics of CRC immune subtypes, which may offer novel targets for personalized immunotherapy.

## Supporting information

S1 Fig Mutational profiles across three immune subtypes. The heatmap shows the top 30 most frequently mutated genes in all the TCGA samples. Y-axis indicates the percentage of patients with at least one mutation in a specific gene.
(TIF)

S2 Fig Associations between immune subtypes and overall survival. Multivariate Cox analyses show the associations of each variables (subtype, age, sex, and stage) with overall survival in the TCGA (**A**) cohort and GSE17536 (**B**) cohort.
(TIF)

S3 Fig Distribution of immunoscores. Boxplots show the distributions of immunoscores in subtypes A, B, and C in the TCGA (**A**) cohort and GSE17536 (**B**) cohort. Immunoscore was calculated using the approach developed by Tang *et al*. [17] and median levels between each two groups were compared by wilcoxon test.
(TIF)
