# Supplementary figures and images for: Identification of immune subtypes and their prognosis and molecular implications in colorectal cancer
Source: PLoS One. 2022 Nov 23;17(11):e0278114. doi: 10.1371/journal.pone.0278114 (PMC9683557; doi:10.1371/journal.pone.0278114)

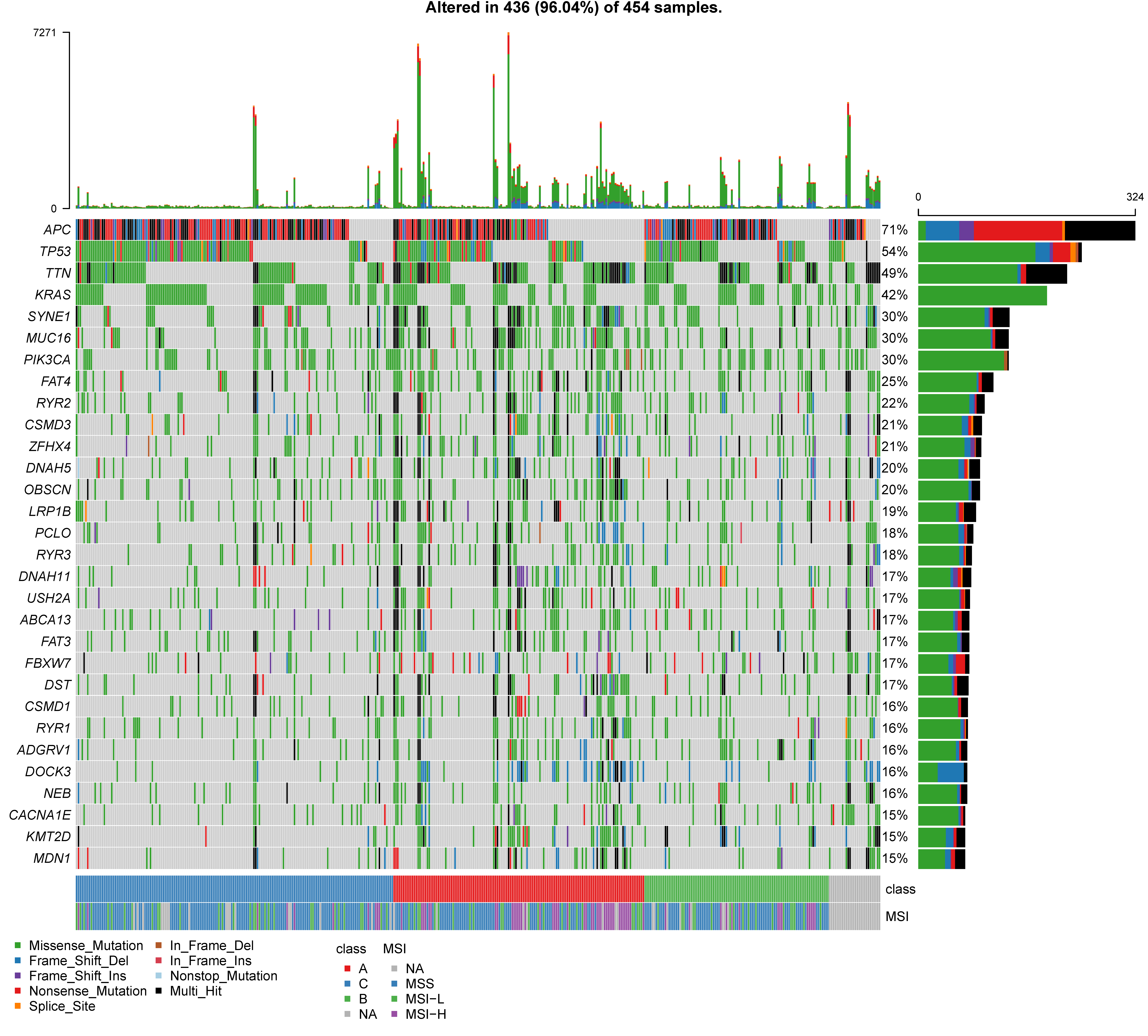

Supplement: S1 Fig — The heatmap shows the top 30 most frequently mutated genes in all the TCGA samples. Y-axis indicates the percentage of patients with at least one mutation in a specific gene. (TIF) [file pone.0278114.s001.tif]

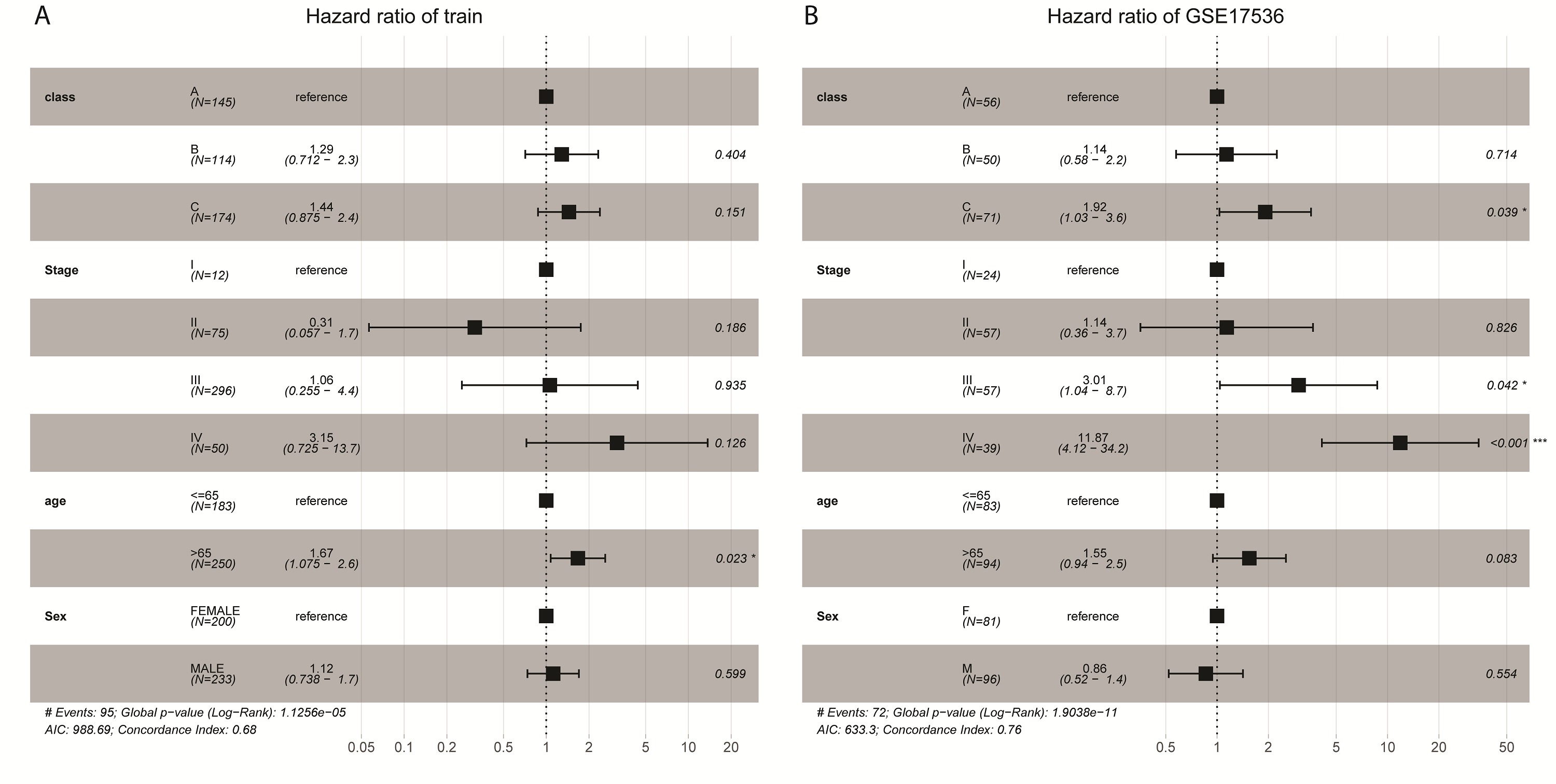

Supplement: S2 Fig — Multivariate Cox analyses show the associations of each variables (subtype, age, sex, and stage) with overall survival in the TCGA (A) cohort and GSE17536 (B) cohort. (TIF) [file pone.0278114.s002.tif]

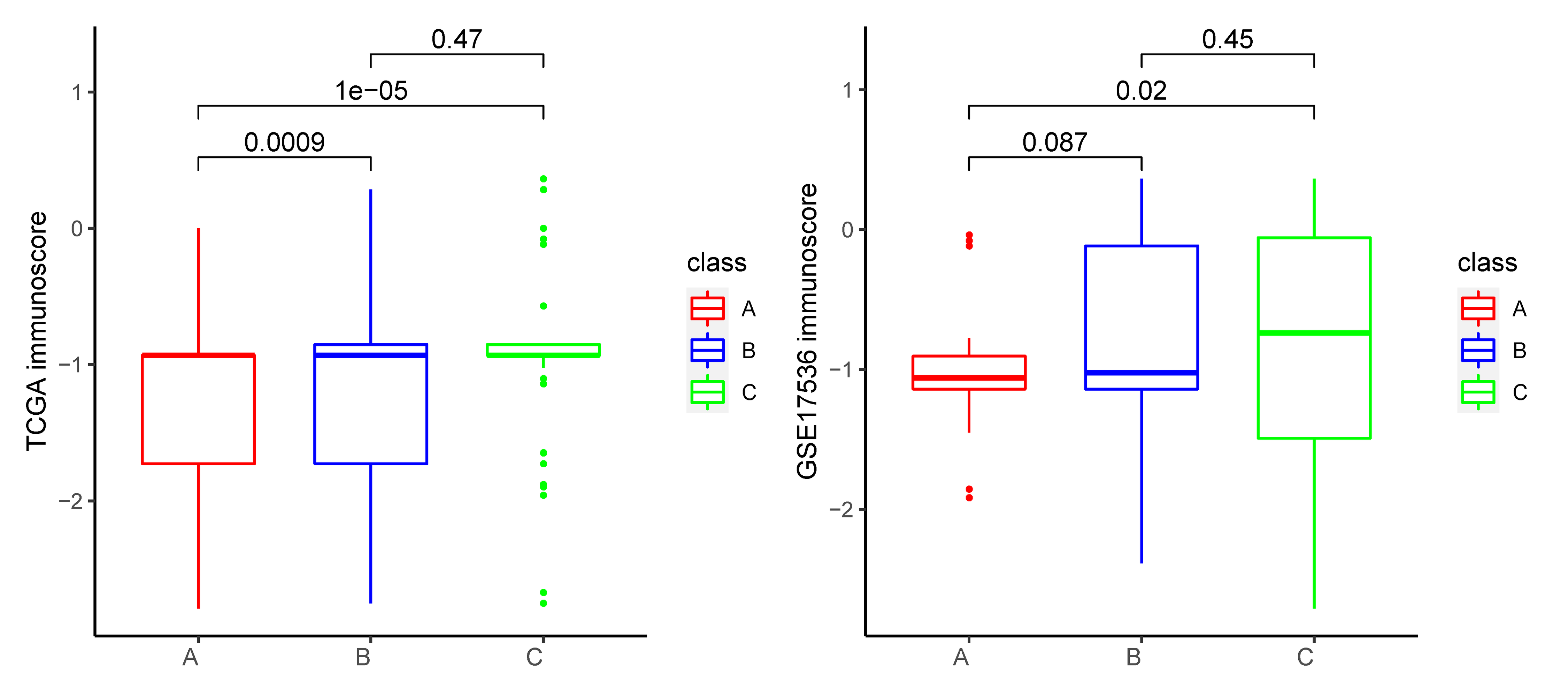

Supplement: S3 Fig — Boxplots show the distributions of immunoscores in subtypes A, B, and C in the TCGA (A) cohort and GSE17536 (B) cohort. Immunoscore was calculated using the approach developed by Tang et al. [17] and median levels between each two groups were compared by wilcoxon test. (TIF) [file pone.0278114.s003.tif]
